# Characterizing the Radioresponse of Pluripotent and Multipotent Human Stem Cells

**DOI:** 10.1371/journal.pone.0050048

**Published:** 2012-12-14

**Authors:** Mary L. Lan, Munjal M. Acharya, Katherine K. Tran, Jessica Bahari-Kashani, Neal H. Patel, Jan Strnadel, Erich Giedzinski, Charles L. Limoli

**Affiliations:** 1 Department of Radiation Oncology, University of California Irvine, Irvine, California, United States of America; 2 Department of Anesthesiology, University of California San Diego, La Jolla, California, United States of America; Instituto Butantan, Brazil

## Abstract

The potential capability of stem cells to restore functionality to diseased or aged tissues has prompted a surge of research, but much work remains to elucidate the response of these cells to genotoxic agents. To more fully understand the impact of irradiation on different stem cell types, the present study has analyzed the radioresponse of human pluripotent and multipotent stem cells. Human embryonic stem (ES) cells, human induced pluripotent (iPS) cells, and iPS-derived human neural stem cells (iPS-hNSCs) cells were irradiated and analyzed for cell survival parameters, differentiation, DNA damage and repair and oxidative stress at various times after exposure. While irradiation led to dose-dependent reductions in survival, the fraction of surviving cells exhibited dose-dependent increases in metabolic activity. Irradiation did not preclude germ layer commitment of ES cells, but did promote neuronal differentiation. ES cells subjected to irradiation exhibited early apoptosis and inhibition of cell cycle progression, but otherwise showed normal repair of DNA double-strand breaks. Cells surviving irradiation also showed acute and persistent increases in reactive oxygen and nitrogen species that were significant at nearly all post-irradiation times analyzed. We suggest that stem cells alter their redox homeostasis to adapt to adverse conditions and that radiation-induced oxidative stress plays a role in regulating the function and fate of stem cells within tissues compromised by radiation injury.

## Introduction

The promise of regenerative medicine has stimulated significant interest in the basic science and clinical application of stem cells. While pluripotent and multipotent stem cells may be used to treat a range of diseases and degenerative disorders, significant work remains to understand their response to a variety of insults within the compromised tissue bed. For cancer patients undergoing radiation or chemotherapy, endogenous stem cells are subjected to harmful agents that are capable of depleting stem cell pools and causing acute and chronic changes in the tissue microenvironment [1], [2]. The initiation of an acute and chronic oxidative stress is one such change caused by irradiation. Alterations in redox homeostasis impact survival, proliferation and cell fate and have a significant impact on the capability of specific tissues to withstand injury [3]. Indeed much of the normal tissue tolerances to irradiation depend on the turnover kinetics of stem cell pools that impact the latency of adverse sequelae. Secondary processes, such as oxidative stress and inflammation that accompany irradiation have a significant impact on the dynamic remodeling of the tissue bed as it adapts to injury [4], [5]. While these processes serve to regulate the regeneration of normal tissue, they also impact the survival, fate and functionality of any transplanted and/or engrafted stem cells. Thus, a more thorough understanding of the response of pluripotent and multipotent stem cells to irradiation may aid efforts to stimulate healing and offset the adverse effects of irradiation on normal tissue.

Our past work has shown that human neural stem cells (hNSCs) derived from embryonic stem cells are markedly sensitive to ionizing radiation, exhibiting a persistent oxidative stress 7 days post-irradiation [6]. Radiation sensitivity was accompanied by apoptosis, cell cycle arrest and elevated metabolic activity that was, in part, responsible for radiation-induced oxidative stress. While acute exposure to clinically relevant doses was found to kill cells, it did not impact the relative proportion of surviving cells undergoing neuronal differentiation nor did it adversely impact the capability of hNSCs to repair highly toxic radiation-induced DNA double-strand breaks. While a handful of studies exist [7]–[10], a more thorough understanding of radiation effects on a variety of stem cells is needed. To expand on previous studies, we have undertaken an extensive analysis of additional pluripotent and multipotent stem cell lines to characterize the consequences of irradiation on these important cell types. Here we report our findings describing the impact of irradiation on embryonic stem cells (ES), induced pluripotent stem cells (iPS) and hNSCs derived from either ES or iPS sources.

## Materials and Methods

### Cell Culture

All procedures utilizing stem cells were in accordance with institutional guidelines approved by the University of California Irvine (UCI) Human Stem Cell Research Oversight (hSCRO) committee (approval number 2007-5629). Cells from the hES cell line H9 (i.e. hESCs, kind gift from Dr. Peter J. Donovan, UC Irvine, originally purchased from WiCell, WI, USA), passages 40 to 70, were cultured on mitotically inactive feeder layers of mouse embryonic fibroblasts (MEF, EMD Millipore, MA, USA). Under these conditions hESCs formed well-defined colonies that were routinely passaged once a week. For subsequent radioresponse analyses, hESCs were passaged on feeder-free, MEF-conditioned media on Matrigel (BD Biosciences, NJ, USA) coated surface.

The iPS cell line (kind gift from Dr. Leslie Lock, UCI hSCRO approval # 2007-5604) was derived from a skin biopsy from a healthy adult female at the University of California, Irvine. IPSCs were subsequently subjected to feeder-free growth on Matrigel (BD Biosciences, NJ, USA) in MEF-conditioned hESC medium. Growth media, which was changed daily, consisted of knockout DMEM with glutamax (Invitrogen, Life Technologies, NY, USA), supplemented with 20% serum replacement (Invitrogen, Life Technologies, NY), 0.1 mM nonessential amino acids (Invitrogen, Life Technologies, NY, USA), 0.1 mM β-mercaptoethanol (Sigma-Aldrich, MO, USA), and basic fibroblast growth factor (bFGF, 4 ng/mL for hESCs and 20 ng/mL for iPSCs, Invitrogen, Life Technologies, NY, USA).

IPS-hNSCs were human neural stem cells derived from iPSCs (induced from a skin biopsy of a normal, healthy individual at University of California, San Diego, UCSD, as described previously, [11]). Derivation and use of iPSCs was approved by UCSD Internal Review Board (IRB, approval ID # 100887). After re-programming of skin cells to iPSCs, colonies were expanded, embryonic bodies were formed and transferred to poly-L-ornithine (20 µg/mL, Sigma-Aldrich, MO, USA) and laminin (5 µg/mL, Sigma-Aldrich, MO, USA) coated petri-dishes for expansion. IPS-hNSCs were sorted using MACS technology (Miltenyi Biotech, Cologne, Germany) to obtain a neural (PSA-NCAM positive) fraction, and were then expanded and sorted for a CD184+/CD24+/CD44−/CD271− fraction using a FACS-ARIA sorter (BD Biosciences, NJ, USA) as described previously [12]. The purified population of proliferating iPS-hNSCs were maintained on EnStem-A neural expansion media (EMD Millipore, MA, USA) containing neurobasal media supplemented with L-glutamine (2 mM, Invitrogen, Life Technologies, NY, USA), bFGF (20 ng/mL, EMD Millipore, MA, USA), B27 and LIF (Leukemia Inhibitory Factor, EMD Millipore, MA, USA). These cells were routinely passaged (1∶2) every other day.

### Irradiation

Exponentially growing cultures were either sham-irradiated or subjected to γ-irradiation (2 or 5 Gy) while anchored in multiwell plates using a ^137^Cs irradiator (J.L. Shepard and Associates Mark I, CA, USA) at a dose rate of 2.2 Gy/min. Immediately after irradiation, cells were placed back in incubators until the time of assay.

### Differentiation

Human ES cells were irradiated and analyzed for qualitative changes in embryoid body (EB) formation. Cells were harvested using collagenase IV (Invitrogen, Life Technologies, NY, USA) and cellular aggregates were transferred to a non-tissue culture treated dish containing ES media without basic fibroblast growth factor (bFGF), which was changed every other day. Images were taken 24 h after irradiation so that any gross morphologic disruption of EB formation could be observed. After 7 days as a floating culture, EBs were transferred to gelatin-coated plates and cultured in the same medium for another 7 days to observe spontaneous differentiation into the different germ layers. These cells were also subjected to marker analysis using β-III-tubulin (Tuj1), smooth muscle actin (SMA) and α-fetoprotein (AFP), which label ectoderm, mesoderm and endoderm, respectively.

### Cell metabolism analysis

The global metabolic state of cells was quantified using a spectrophotometric assay based on cleavage of tetrazolium salt (XTT; Sigma–Aldrich, MO, USA). Metabolically active cells reduce XTT via trans-plasma membrane electron transport coupled with mitochondrial oxidoreductase activity [13], [14]. The reduced XTT can be measured at 492 nm. Therefore, the XTT assay provides an indirect measure of global cellular metabolism. Cells were incubated for 30 min in phenol-free medium containing XTT and the activating reagent (phenazine methosulfate; Sigma–Aldrich, MO, USA), after which absorbance was measured immediately using a microplate reader (SynergyMx; BioTek, VT, USA). These assays preceded the analysis of cell number by DNA content described below, such that the analyses of metabolism and survival were performed on the same cells within the same sample wells. In this way, we were able to directly assess the metabolic state of stem cells surviving irradiation.

### Cell survival analysis

Cell survival was determined using SYBR green fluorescence to quantify DNA following protocols described previously [6]. For survival assays, hESCs were seeded at 65,000 cell/well, iPS cells were seeded at 84,000 cells/well and iPS-hNSCs were seeded at 50,000 cells/well. Cells were then irradiated at 0, 2, or 5 Gy. One extra plate was seeded to define the seeding density since hESCs are maintained as colonies and cut into pieces, which are subsequently transferred for passaging. Cells were grown for 5 days, washed with PBS, and incubated at −80°C overnight. Cells were then lysed using M-PER (Mammalian Protein Extraction Reagent; Thermo Scientific, IL, USA) containing 2.5× SYBR Green I (Invitrogen, Life Technologies, NY, USA). SYBR Green I was excited at 497 nm and the fluorescence was measured at 520 nm on a microplate reader (SynergyMx; BioTek, VT, USA) and compared to a standard curve to yield actual cell counts.

### Measurement of apoptosis

An antibody against the 85-kDa caspase cleavage product of poly(ADP-ribose) polymerase (PARP; Invitrogen, Life Technologies, NY, USA) was used to quantify apoptotic cells in irradiated and unirradiated hESCs. All procedures were in accord with the manufacturer's protocol. Briefly, control and irradiated cells (5 Gy) were harvested at various time points (6, 12, 24, and 48 h), rinsed twice in PBS, and fixed with 4% paraformaldehyde (PFA; Electron Microscopy Sciences, PA, USA) followed by a 30-min incubation at 4°C with the anti-PARP FITC-conjugated antibody (Invitrogen). After antibody incubation, cells were washed three times and resuspended in PBS for fluorescence-activated cell sorting (FACS) analysis using Guava EasyCyte (Guava Technologies/EMD Millipore, MA, USA).

Radiation-induced apoptosis was also quantified by the annexin V–FITC apoptosis kit (BD Biosciences, NJ, USA) following the manufacturer's recommendations. Annexin V is a Ca^2+^-dependent phospholipid-binding protein that has a high affinity for phosphatidylserine residues externalized to the outer leaflet of the plasma membrane. At the indicated times after irradiation, cells were washed in isotonic buffer and then resuspended in binding buffer, incubated with the annexin V–FITC conjugate, and analyzed by FACS.

### Cell-cycle analysis

At various times (6, 12, 24, and 48 h) post-irradiation, exponentially growing cultures of hESCs were fixed with 4% PFA and stored in 10% sucrose in sterile PBS. Before fixation, cells were incubated with 20 µM bromodeoxyuridine (BrdU; Sigma–Aldrich, MO, USA) for 1 h. On the day of assay, cell-cycle analysis was performed using a BrdU flow cytometry kit (BD Biosciences, NJ, USA) following the manufacturer's instructions. Anti-BrdU–FITC- (1∶250; BD Biosciences, NJ, USA) and propidium iodide (Sigma–Aldrich, MO, USA) stained cells were analyzed by FACS, and FCS Express software (version 3.0) was used to derive the fraction of cells distributed throughout various phases of the cell cycle.

### γ-H2AX analysis

To quantify the level of H2AX phosphorylation after irradiation, immunocytochemistry, FACS analysis, and γ-H2AX foci enumeration were carried out. Cells were fixed and permeabilized in preparation for FACS analysis following the instructions provided in the H2AX FACS kit (Upstate–Chemicon, EMD Millipore, MA, USA) at various post-irradiation time points (0 h, 20 min, and 1, 2, 6, 24 and 48 h). Negative and positive controls (γ-irradiated) for γ-H2AX were included for comparison. To compute the relative levels of H2AX phosphorylation, the ratio of ungated fluorescence means (test cells/untreated controls) was used. In parallel, hESCs were fixed using 4% PFA for 20 min, followed by incubation with chilled (−20°C) methanol for 30 min on slides at various times as described above, permeabilized using 0.1% Triton X-100 and stained with anti-γ-H2AX (1∶100; Abcam, MA, USA). Alexa Fluor-488 (1∶150; Invitrogen, Life Technologies, NY, USA) was used as the secondary antibody, and cells were counterstained with DAPI to identify γ-H2AX-foci positive nuclei by fluorescence microscopy (Nikon Eclipse TE2000-U, Nikon Instruments, NY, USA). After fluorescence microscopy, images were uploaded to the Nikon Elements AR (version 3.0) image analysis program. Multichannel threshold and an automated binary counting module were used to quantify the number of nuclei (DAPI fluorescence) and the number of γ-H2AX foci in each nuclei as described in detail previously [6]. Once optimal settings were configured, this program facilitated the automated counting of fluorescent peaks (green γ-H2AX foci) within the selected region of interest (ROI; blue DAPI-fluorescent nuclei). All software configurations were kept constant for all groups (irradiated and sham-irradiated) across each of the time points analyzed.

### Measurement of oxidative stress

The detection of intracellular ROS and RNS was based on the ability of live cells to oxidize fluorogenic dyes to their corresponding fluorescent analogs. Exponentially growing hESCs were treated for 45 min at 37°C with the ROS/RNS-sensitive dye 5-(and-6)-chloromethyl-2′, 7′-dichlorodihydrofluorescein diacetate (CM-H_2_DCFDA; 5 µM; Invitrogen, Life Technologies, NY, USA), the nitric oxide-sensitive dye 4-amino-5-methylamino-2′, 7′-difluorofluorescein diacetate (DAF; 5 µM; Invitrogen, Life Technologies, NY, USA), or the superoxide-sensitive dye MitoSOX (0.5 µM; Invitrogen, Life Technologies, NY, USA). Immediately after dye incubation (45 min), cells were harvested and analyzed by flow cytometry. For each post-irradiation time, measurements of ROS/RNS (CM-H_2_DCF), superoxide (MitoSOX), or nitric oxide (DAF) in irradiated and sham-irradiated control cultures were performed in parallel. All measurements were carried out in triplicate or quadruplicate and were derived from independently irradiated cultures of hESCs.

### Immunocytochemistry

Immunocytochemical studies used both monoclonal and polyclonal primary antibodies. The monoclonal primary antibodies comprised anti-Oct4 (Rabbit, 1∶250, Abcam, MA,USA), anti-SSEA-4 (Mouse, 1∶250, Abcam, MA, USA), anti-SMA (Mouse, 1∶1000, Chemicon, EMD Millipore, MA, USA), anti-AFP (Mouse, 1∶100, R&D Systems, MN, USA), anti-Tuj1 (Mouse, 1∶500, Covance, NJ, USA). The secondary antibodies (1∶200 dilution) included anti-mouse and anti-rabbit IgG conjugated to Alexa Fluor (488 and 594, Invitrogen, Life Technologies, MA, USA).

### Statistical analysis

Data are represented as the means ± SEM of three to six individual experiments and the level of significance was assessed by Student's *t* test (Prism data analysis software, version 3.0). Statistical significance was assigned at a *P* value≤0.05.

## Results

### Irradiation and cell fate

**Figure 1:**
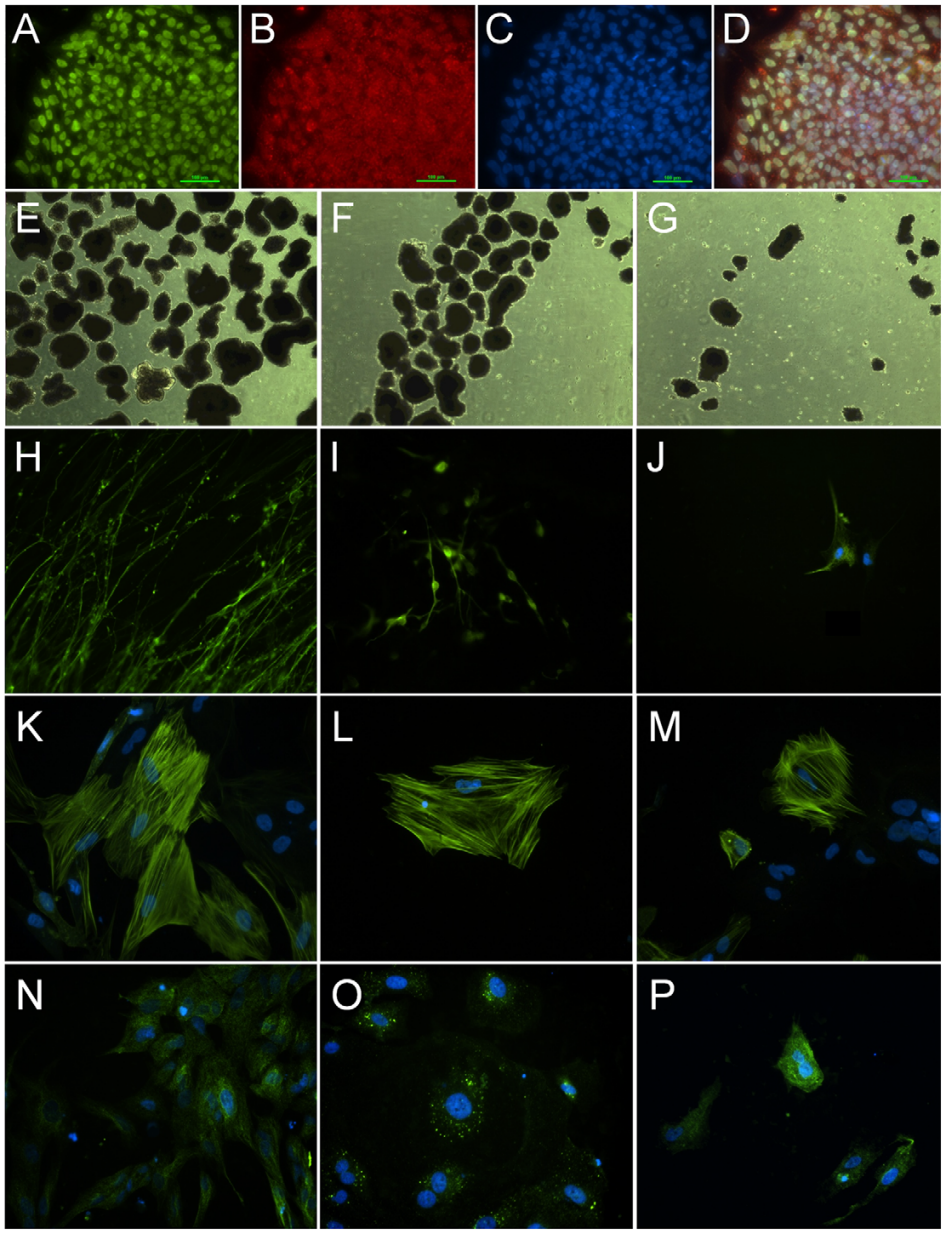
Pluripotent and early germ-layer markers in hES cells. Immunocytochemical analysis of hES cells reveals strong expression and co-localization of the pluripotent transcription factors (Octamer-4, Oct-4, A) and Stage Specific Embryonic Antigen (SSEA-4, B) counter-stained with 4′,6′-diamidino-2-phnylindole (DAPI, C) shown as merged image (D). Phase contrast images taken 24 after 0, 2 and 5 Gy (E–G, respectively) show the effect of irradiation on embryoid body formation. To gauge the impact of irradiation on germ layer differentiation (H–P), an ectodermal marker β-III-Tubulin (Tuj1, H–J), a mesodermal marker α-Smooth Muscle Actin (α-SMA, K–M) and an endodermal marker α-fetoprotein (AFP, N–P) were used after 0 (H,K,N), 2 (I,L,O) and 5 Gy (J,M,P) respectively. Scale bars, 100 µm (A–D), 50 µm (E–G) and 10 µm (H–P).

To determine the impact of irradiation on the capability of hES cells to form embryoid bodies and differentiate into the three basic germ layers, hES cells were irradiated and analyzed at various times after irradiation. The pluripotent markers Oct-4 and SSEA-4 showed strong expression and co-localization in unirradiated and undifferentiated ES cells (Fig. 1, A–D). Irradiation of embryoid bodies caused disruptions to their morphology, largely due to radiation-induced cell death that resulted in smaller and more fragmented structures (Fig. 1, E–G). Despite the radiation-induced cell loss that depleted cells during the course of differentiation, irradiation did not preclude commitment of ES cells to the 3 primary germ layers. Cells analyzed 1 week after irradiation were found to express Tuj1, α-SMA and AFP, markers that are typically expressed in the ectoderm, mesoderm and endoderm germ layers respectively (Fig. 1, H–P).

### Survival, metabolic activity and cell cycle

**Figure 2:**
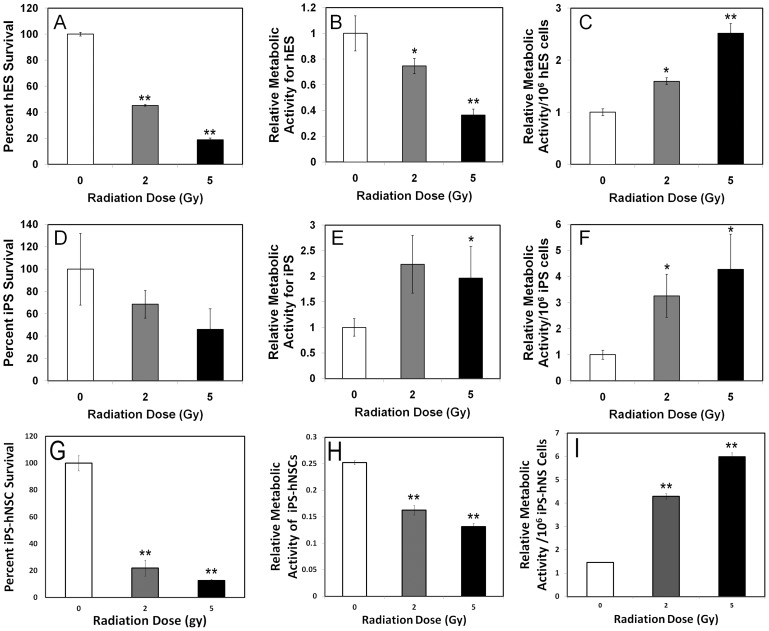
Cell survival and metabolic activity in irradiated hES, iPS and iPS-hNSCs. Exponentially growing hESCs, iPSCs and iPS-hNSCs were seeded at densities of 65,000, 84,000 and 50,000 cells/well respectively. Cell counts were quantified by SYBR green-based fluorescence. The number of stem cells exhibited a dose-dependent decline 5 days after irradiation (A, D and G). The global metabolic activity measured in the same cells using the XTT assay showed a similar dose-dependent decline for hESCs (B) and iPS-hNSCs (H). An opposite pattern was observed for iPSCs (E). Normalizing the global metabolic activity per million cells revealed a dose-dependent increase in cellular metabolism for all cell types (C, F, I). Data expressed as the Mean ± S.E.M. of 3–4 independent observations compared to unirradiated controls. *, *P*<0.05 and **, *P*<0.01.

To determine the relative sensitivity of stem cells to irradiation, ES, iPS and iPS-hNSC were irradiated and analyzed for changes in survival and metabolic activity 5 days later. Reductions in survival were dose-dependent and significant (p<0.05), and qualitatively similar trends were found in each of the cell lines tested. Human ES and iPS-derived hNSCs were more sensitive than pluripotent iPS cells, as 5 Gy doses reduced survival to 20 versus 43% respectively (Fig. 2, A, D, G). Changes in metabolic activity after irradiation were different between the cell types analyzed. While ES and iPS-derived hNSCs showed dose dependent reductions in XTT absorbance (Fig. 2, B and H), iPS cells showed increased XTT absorbance that was less dependent on dose (Fig. 2E). Interestingly, when metabolic activity was normalized to the number of surviving cells, radiation was found to increase the metabolic activity of the irradiated survivors for all cell types analyzed (Fig. 2C, F and I). Radiation-induced increases in normalized metabolic activity were found to be significant for each cell type and at all doses, suggesting the capability of stem cells to alter their metabolic profiles in response to genotoxic stress [15].

**Figure 3:**
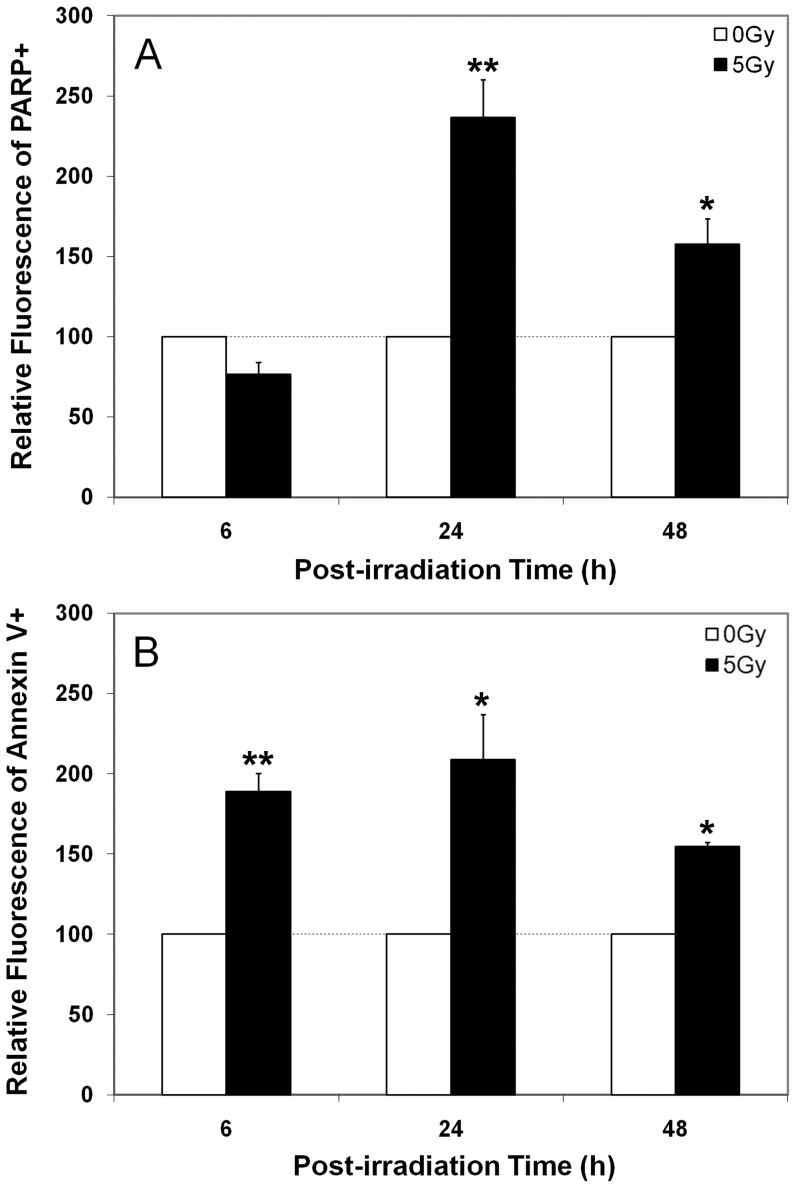
Radiation-induced apoptosis in hES cells. Early stage (PARP cleavage, A) or latter stage (annexin V binding, B) apoptosis was increased significantly at nearly all times analyzed (2, 24, 48 h) following a 5 Gy dose. Data expressed as the Mean ± S.E.M. of 3 independent observations compared to unirradiated controls. *, *P*<0.01 and **, *P*<0.001.

Past work has shown that hNSCs were sensitive to radiation-induced apoptosis, and the present study sought to determine if hESC showed similar levels of apoptosis following irradiation. Analysis of irradiated hESCs showed marked and significant (p<0.05) increases in apoptosis at all times analyzed over a 48 h post-irradiation interval (Fig. 3). Based on 2 reliable measures of apoptosis, a 5 Gy dose was found to increase the number of apoptotic cells 1 and 2 days after exposure (Fig. 3). Compared to sham-irradiated controls, detection of the caspase mediated PARP cleavage product in hESCs cells peaked (>2-fold) at 24 h (Fig. 3A), while annexinV positive cells showed a consistent increase (1.5–2 fold) over a 6–24 h timeframe (Fig. 3B).

**Figure 4:**
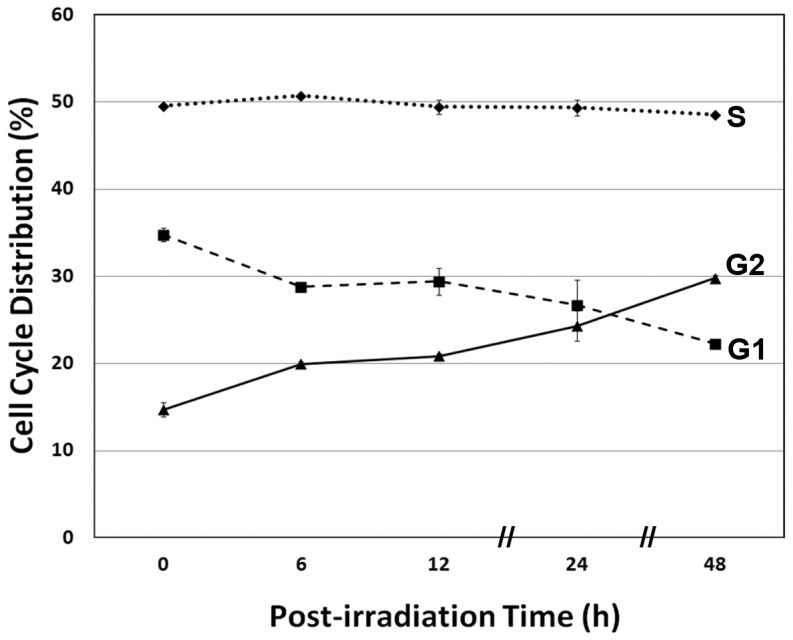
Analysis of cell cycle progression in irradiated hES cells. Exponentially growing cultures of hES cells exposed to 5 Gy showed an increase in the proportion of cells in G2/M and a decrease in the proportion of cells in G1. Data expressed as the Mean ± S.E.M. of 3 independent observations compared to unirradiated controls.

Over a similar time course, a 5 Gy dose was found to elicit a relatively modest inhibition of cell cycle progression in hESCs (Fig. 4). The distribution of cells throughout the cell cycle was analyzed by FACS following pulse-labelling with BrdU and propidium iodide staining. The fraction of cells in G2/M was found to increase steadily at the expense of cells in G1 (Fig. 4). Over a 2-day post-irradiation interval, the number of cells at G2/M increased ∼25% by 6 h, and nearly doubled by 48 h, representing 30% of the surviving irradiated population (Fig. 4).

### DNA damage and repair

**Figure 5:**
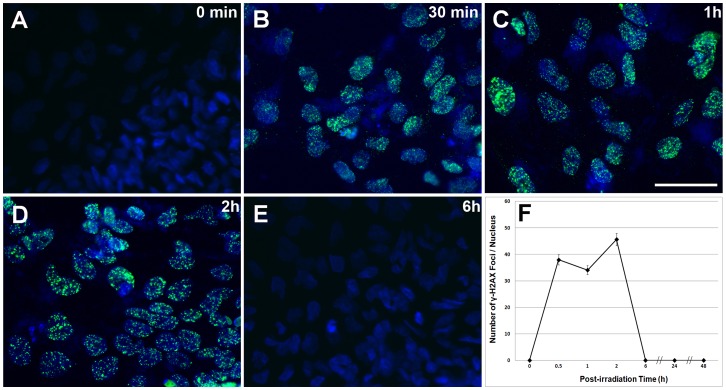
Repair of DNA double-strand breaks (DSB) in irradiated hES cells. The repair kinetics of DNA DSBs was monitored by the formation and removal of γ-H2AX nuclear foci (green) in cells counterstained with the nuclear dye DAPI (blue, A–E). Immunocytochemical analysis of hESCs exposed to 5 Gy irradiation over a 6 h interval showed typical yields of γ-H2AX nuclear foci from 0.5–2 h (B, C and D) as compared to 0 Gy controls (A). The number of DSBs dropped rapidly after 2 h (E) indicating functional DSB repair. Peak yields of γ-H2AX foci occurred at 2 h before falling to baseline levels at 6 h post-irradiation (F). Data expressed as the Mean ± S.E.M. of 3–6 independent observations. Scale bars, 5 *µm* in A–E.

In prior work analyzing DNA repair in hNSCs [6], irradiated cells were analyzed for the appearance and subsequent removal of intranuclear γ-H2AX foci, used as a marker for the repair kinetics of DNA DSBs. We decided to use a similar strategy to analyze DSB repair kinetics in hESCs, as the majority of past reports analyzing DNA repair in hESCs did not quantify the multiplicity of intranuclear γ-H2AX foci over time. Thus, to evaluate the DNA repair capacity of hESCs, the formation and removal of DNA repair foci was assessed over a 6 h post-irradiation interval. As expected, hESCs cells subjected to a 5 Gy dose of γ-rays showed a rapid and marked rise in the number of cells positive for γ-H2AX nuclear foci (Fig. 5). The number of foci positive cells increased during the first 2 h following irradiation before dropping to baseline levels by 6 h (Fig. 5). The multiplicity of nuclear foci followed similar kinetics, with peak foci levels observed between 1–2 h after irradiation, falling to baseline levels thereafter.

### Oxidative Stress

**Figure 6:**
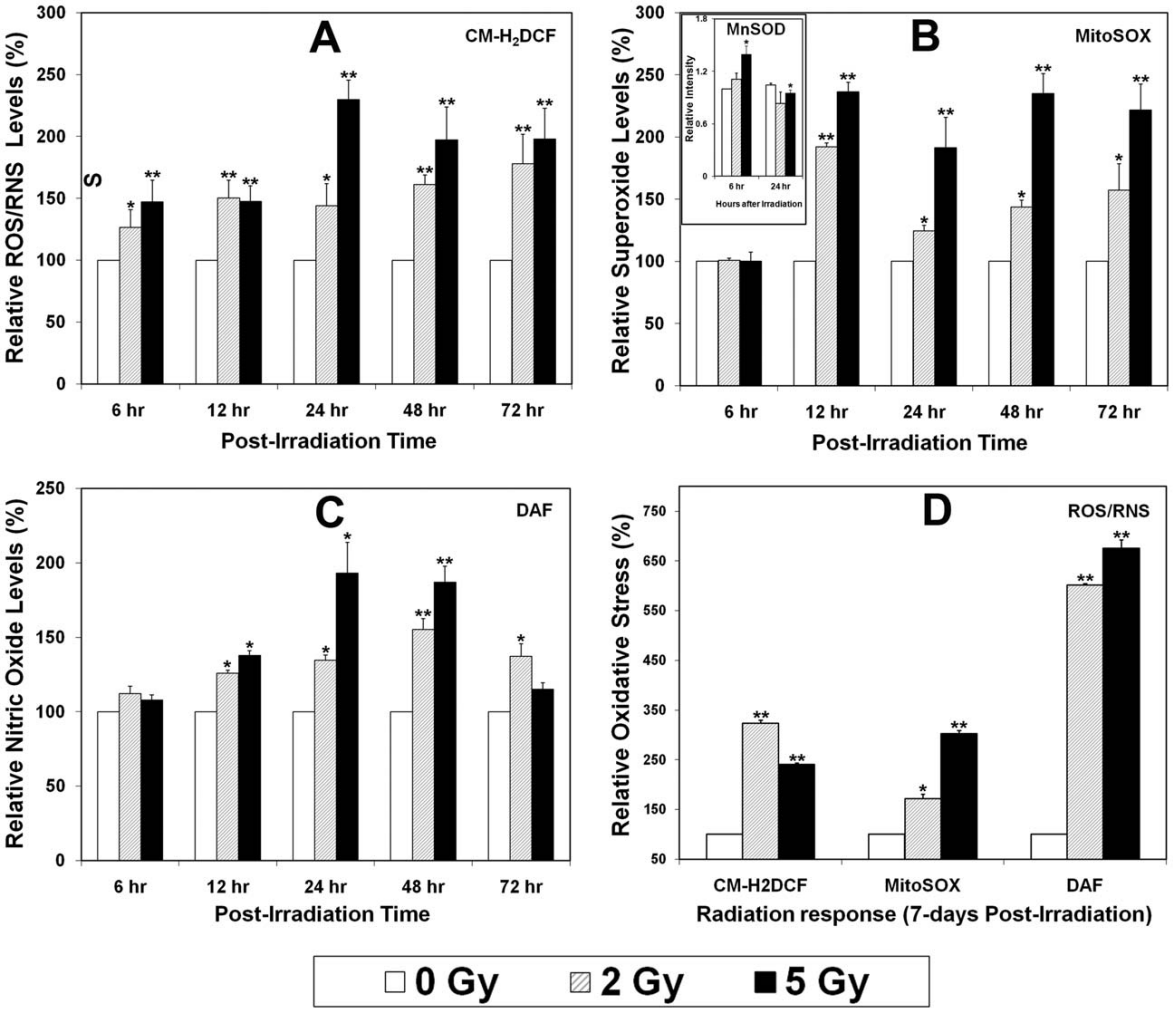
Reactive oxygen and nitrogen (ROS and RNS) species in irradiated hES cells. Exponentially growing cultures of hESCs were γ-irradiated and assayed for reactive species using the ROS/RNS-sensitive dye CM-H_2_DCF (A), the superoxide-sensitive dye MitoSOX (B), or the nitric oxide-sensitive dye DAF (C). FACS analyses of irradiated hESCs showed increased ROS/RNS, superoxide and nitric oxide over a 72 h post-irradiation interval. Increased levels of these reactive species were in general, found to be dose-responsive at most time points analyzed (A–C). Relative levels of Mn-SOD (as detected by western blotting) correlated inversely with superoxide levels (MitoSox) at a dose of 5 Gy (insert, B). Longer-term analyses conducted 7 days post-irradiation (D) revealed persistent and dose-responsive increases in oxidative stress. Data expressed as the Mean ± S.E.M. of 3–6 independent observations and all values are normalized to controls FACS analyzed on the same day. *, *P*<0.05 and **, *P*<0.01.

Previous studies from us and others have shown that NSCs from rodents and humans, along with bone and muscle progenitors exhibit a persistent oxidative stress following irradiation [6], [16]–[20]. To determine whether irradiated hESCs might exhibit a similar response following irradiation, cells were analyzed for oxidative stress for 1 week following exposure using a suite of fluorogenic dyes. Overall trends showed that irradiated hESCs exhibited acute and chronic increases in oxidative stress. Shorter-term measurements taken periodically over the course of 3 days revealed significant (p<0.05) increases in ROS/RNS (Fig. 6A), superoxide (Fig. 6B) and nitric oxide (Fig. 6C) in irradiated hESCs compared to controls. Increased levels of reactive species at various time points were largely dose-responsive, reaching peak levels by 24 h that remained elevated over 2 days (Fig. 6A–C). Each of the fluorogenic dyes showed qualitatively similar responses, with elevated levels of reactive species varying from 1.2–2.5-fold over sham-irradiated controls normalized to unity. Importantly, oxidative stress did not abate in irradiated cultures of hESCs, as each of the dyes showed enhanced levels of reactive species up to 7 days post-irradiation (Fig. 6D). While dose-responsive changes were less evident at this protracted post-irradiation time point, the magnitude of elevated ROS/RNS and superoxide were comparable (1.5–2.5 fold increases) to those found at earlier times. Nitric oxide levels were however, markedly elevated 1-week following irradiation, rising 5–6 fold over unirradiated controls (Fig. 6D). Radiation-induced oxidative stress in hESCs cells was also accompanied by corresponding changes in manganese superoxide dismutase (MnSOD). The mitochondrial isoform of SOD was induced by irradiation (Fig. 6B, inset), and found to be relatively high at 6 h, a time at which superoxide levels were minimal. By 24 h, MnSOD levels were at or below unirradiated controls, a time at which superoxide levels were elevated compared to controls (Fig. 6B).

## Discussion

Human stem cells exposed to ionizing radiation respond by engaging DNA damage responsive pathways not unlike other mammalian cells. Past work has demonstrated the sensitivity of hESCs to irradiation, and the capability of these cells to repair DNA damage and engage the G2/M checkpoint in response to this damage [7]–[10]. Consistent with these past findings, irradiated hESCs exhibited early apoptosis that was exacerbated in the absence of a G1/S checkpoint, which contributes to the sensitivity of these cells to many genotoxic agents (Figs. 3, 4). Many of the radiation-induced responses found for hESCs were qualitatively similar to those reported by us in an earlier study analyzing the radioresponse of hNSCs [6]. Radiation-induced depletion of ES cells disrupts the morphologic integrity of embryoid bodies, but does not appear to prevent ES cells from committing to the basic germ cell lineages required for further development (Fig. 1). Less known however, is if/how irradiation might impact the further differentiation of irradiated ES cells, a topic beyond the scope of the current study.

Survival and metabolic measurement of irradiated hESCs, iPS and iPS-derived hNSCs all showed qualitatively similar trends (Fig. 2). Dose-responsive reductions in cell counts were generally paralleled by reductions in metabolic activity, with the exception of the iPS cells (Fig. 2E). Sensitivity of hESCs and hNSCs could in part be explained by radiation-induced apoptosis transpiring within the first few days following irradiation, Fig. 3, [6]. Repair of DNA double-strand breaks (DSBs) was not compromised, as the removal of intranuclear γ-H2AX foci was completed by 6 h following a 5 Gy dose. While repair of these lesions was complete at this time, the removal kinetics were somewhat atypical, as the multiplicity of intranuclear foci remained elevated for several hours after irradiation, before returning to baseline levels over the subsequent 4 h (Fig. 5). Interestingly, hNSCs exhibited similar DSB repair kinetics, where high levels of intranuclear foci persisted for several hours before returning to basal levels [6]. Redistribution of chromatin containing these breaks to repair factories at the nuclear periphery may explain the delayed removal kinetics [21], but other explanations are possible.

For the fraction of cells surviving irradiation, metabolic activity increased with dose (Fig. 2C, F, I). While the precise causes of this are uncertain, an upregulation of metabolism in response to irradiation may reflect a compensatory response of stem cells to genotoxic stress. The capability to modulate redox homeostasis may also reflect a more general strategy to adjust cellular energy requirements following adverse stimuli in efforts to promote survival [3], [22], [23]. Radiation-induced alterations in metabolism likely underlie the development of oxidative stress over acute and extended post-irradiation times. Intracellular pro-oxidants were elevated significantly over unirradiated controls during the 1-week interval following acute exposures (Fig. 6). For the spectrum of reactive species analyzed, the persistence and the dose responsive nature of the changes suggest that radiation-induced oxidative stress was not transient, but rather, a more chronic condition capable of altering the microenvironmental niche and the long-term functionality of stem cells [24], [25] [26].

Present findings advance much of our past work aimed at characterizing the radioresponse of specific stem cell types. As the number of cancer survivors and patients receiving radiotherapy continues to grow, the need to understand the basic science of radiation effects in stem cell takes on greater importance. The use of stem cells in regenerative medicine also imparts the need to more fully understand the stress response of pluripotent and multipotent stem cells. Endogenous and/or transplanted stem cells important to the eventual recovery of the irradiated tissue bed may be subject to repeat irradiation, depending on treatment protocols or disease recurrence. Studies were thus undertaken to define the important cellular processes that might compromise the functionality of these cells in the irradiated brain or other tissues. Present data indicate that irradiated stem cells respond similarly as other mammalian cell types, showing increased apoptosis, inhibition of cell-cycle progression and the capability to repair DNA damage [6], [19]. The foregoing processes define important survival parameters and characterize the DNA damage response of stem and non-stem cells alike. Irradiation also increased the metabolic activity of surviving cells and elevated oxidative stress over a 1-week post-irradiation interval. Changes in redox state may be responsible for determining the fate of stem cell differentiation, and highlight the potential importance of radiation-induced oxidative stress as a biochemical mechanism for altering stem cell functionality in tissues compromised by prior irradiation.
